# Sonoenzymatically Triggered Cascading Degradation of Bioresorbable Materials for On‐Demand Transient Triboelectric Implants

**DOI:** 10.1002/adma.73921

**Published:** 2026-07-08

**Authors:** Jinsong Kim, Dong‐Min Lee, Youngwook Chung, Byung‐Joon Park, Hyeon Mo, Jong Won Seon, Han‐Yup Yum, Bosung Kim, Byung‐Ok Choi, Sang‐Woo Kim

**Affiliations:** ^1^ Department of Materials Science and Engineering Yonsei University Seodaemun‐gu Seoul Republic of Korea; ^2^ Center for Human‐oriented Triboelectric Energy Harvesting Yonsei University Seoul Republic of Korea; ^3^ Center for Bio‐Integrated Electronics Northwestern University Evanston Illinois USA; ^4^ Querrey Simpson Institute for Bioelectronics Northwestern University Evanston Illinois USA; ^5^ Department of Neurology, Samsung Medical Center Sungkyunkwan University School of Medicine Gangnam‐gu Seoul Republic of Korea; ^6^ Cell and Gene Therapy Institute Samsung Medical Center Gangnam‐gu Seoul Republic of Korea; ^7^ Department of Health Sciences and Technology Samsung Advanced Institute for Health Sciences & Technology (SAIHST) Gangnam‐gu Seoul Republic of Korea

**Keywords:** bioresorbable materials, enzymatic degradation, nanocarriers, transient electronics, ultrasound‐driven energy harvesting

## Abstract

Bioresorbable materials provide temporary biomedical support but often generate wear debris during degradation, leading to immune responses and long‐term complications. Here, we present a sonoenzymatic activation (SEA) materials strategy for on‐demand, cascading in vivo degradation of bioresorbable systems. By coupling ultrasound‐triggered mechanical disintegration with enzyme‐catalyzed molecular degradation, we design a bioresorbable composite embedding enzyme‐encapsulated, biocompatible metal‐organic frameworks (BCEM). Ultrasound‐induced cavitation enables temporally controlled enzyme release, which cleaves ester bonds in the polycaprolactone matrix. In vivo, BCEM undergoes rapid mechanical fragmentation within 15 min under medically relevant ultrasound (20 kHz, 1.0 W cm^−^^2^), followed by enzyme‐assisted boosted degradation that reduces the persistence of residues over the 14‐day observation period. This SEA strategy establishes a general materials platform for bioresorbable implants with programmable lifetimes and non‐invasive elimination.

## Graphical abstract

Our SEA strategy provides an efficient and biosafe approach for the on‐demand removal of implanted bioresorbable materials through cascading degradation that couples ultrasound‐triggered rapid mechanical disintegration with subsequent enzymatic degradation. This approach paves the way for the clinical translation of bioresorbable implant systems, enabling safe and controllable in vivo transience. Created in BioRender. Kim, J. (2026) https://BioRender.com/nfxykal.

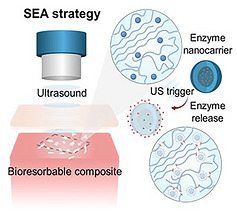

## 1 Introduction

Bioresorbable materials are widely incorporated into implants to provide temporary therapeutic support while eliminating the need for secondary removal through natural resorption. However, their intrinsically slow and incomplete degradation often generates wear particles and debris, which can provoke chronic inflammation and long‐term complications, such as thrombosis [1, 2, 3, 4, 5, 6]. These limitations highlight the need for strategies that enable rapid and controllable degradation of bioresorbable materials after the therapeutic period.

Active transience systems, which trigger material degradation in response to external stimuli, offer a promising route to address this challenge [7, 8]. Various activation strategies, including light [9], thermal [10], and ultrasound [11], have been explored. While light and thermal approaches are limited in vivo by shallow penetration depths and potential tissue damage, ultrasound is clinically established and enables deep, non‐invasive energy delivery. However, ultrasound alone induces only mechanical disintegration and cannot achieve molecular‐level degradation. Enzymatic degradation provides a complementary mechanism by enabling catalytic hydrolysis under physiological conditions with biocompatible by‐products [12]. Yet, the direct administration of enzymes and drugs may induce immune and allergic responses [13, 14], motivating the use of encapsulation within nanocarriers such as liposomes [15], polymeric nanoparticles [16], and metal–organic frameworks [17]. Although these nanocarrier systems have been widely explored for therapeutic applications, including cancer therapy, immunotherapy, and genome editing [15, 16, 17, 18], their use to promote the in vivo removal of bioresorbable implant materials remains largely underexplored.

Here, we introduce a sonoenzymatic activation (SEA) materials strategy that enables cascading degradation by integrating ultrasound‐triggered mechanical disintegration with enzyme‐catalyzed hydrolysis. We design a bioresorbable composite incorporating enzyme‐encapsulated, biocompatible metal–organic frameworks (BCEM), which simultaneously act as enzyme reservoirs and acoustic impedance mismatching elements. Upon ultrasound exposure, BCEM undergoes rapid mechanical fragmentation, followed by controlled enzyme release that catalyzes molecular‐scale degradation. In vitro and in vivo studies demonstrate that ultrasound‐triggered BCEM (1.0 W cm^−^^2^, 15 min) exhibits boosted degradation by minimizing the residue size and its persistence over the 14‐day observation period, whereas enzyme‐free controls remain fragmented, with no apparent further degradation. Comprehensive biocompatibility assessments confirm the biosafety of the degradation products. Furthermore, integration of BCEM into a biomedical implant demonstrates ultrasound‐programmable functional lifetimes, establishing SEA as a general materials platform for on‐demand, clinically adaptable bioresorbable systems.

## 2 Results and Discussion

### 2.1 BCEM for SEA Strategy

To demonstrate the SEA strategy, we utilized polycaprolactone (PCL), lipase enzyme, and biocompatible metal‐organic framework (bio‐MOF) nanocarriers. PCL is FDA‐approved in scaffolds and sutures and has a slow degradation rate (∼10% mass loss within 4 weeks) [19, 20, 21]. Lipase is widely known as an effective esterase degrading PCL (Figure S1) [22, 23]. Compared with other nanocarriers (e.g., liposomes), bio‐MOF nanocarriers are selected for their dual functionality: providing stable enzyme encapsulation [24] and facilitating ultrasound‐triggered mechanical disintegration through internal air fractions [11] within their pores. Here, zeolitic imidazolate framework‐8 (ZIF‐8) was selected among various bio‐MOF candidates for its specific advantage. Generally, releasing enzymes directly through the MOF pores is challenging due to the narrow pore size. The co‐synthesis method is widely employed to encapsulate enzymes within MOFs, enabling their immobilization and thereby improving reusability and stability [25]. Therefore, enzyme release generally requires dissolution of the MOF structure, which often involves pH changes (pH 5–6) to break coordination bonds between metal ions and organic ligands, which are incompatible with physiological conditions (pH 7).

**FIGURE 1:**
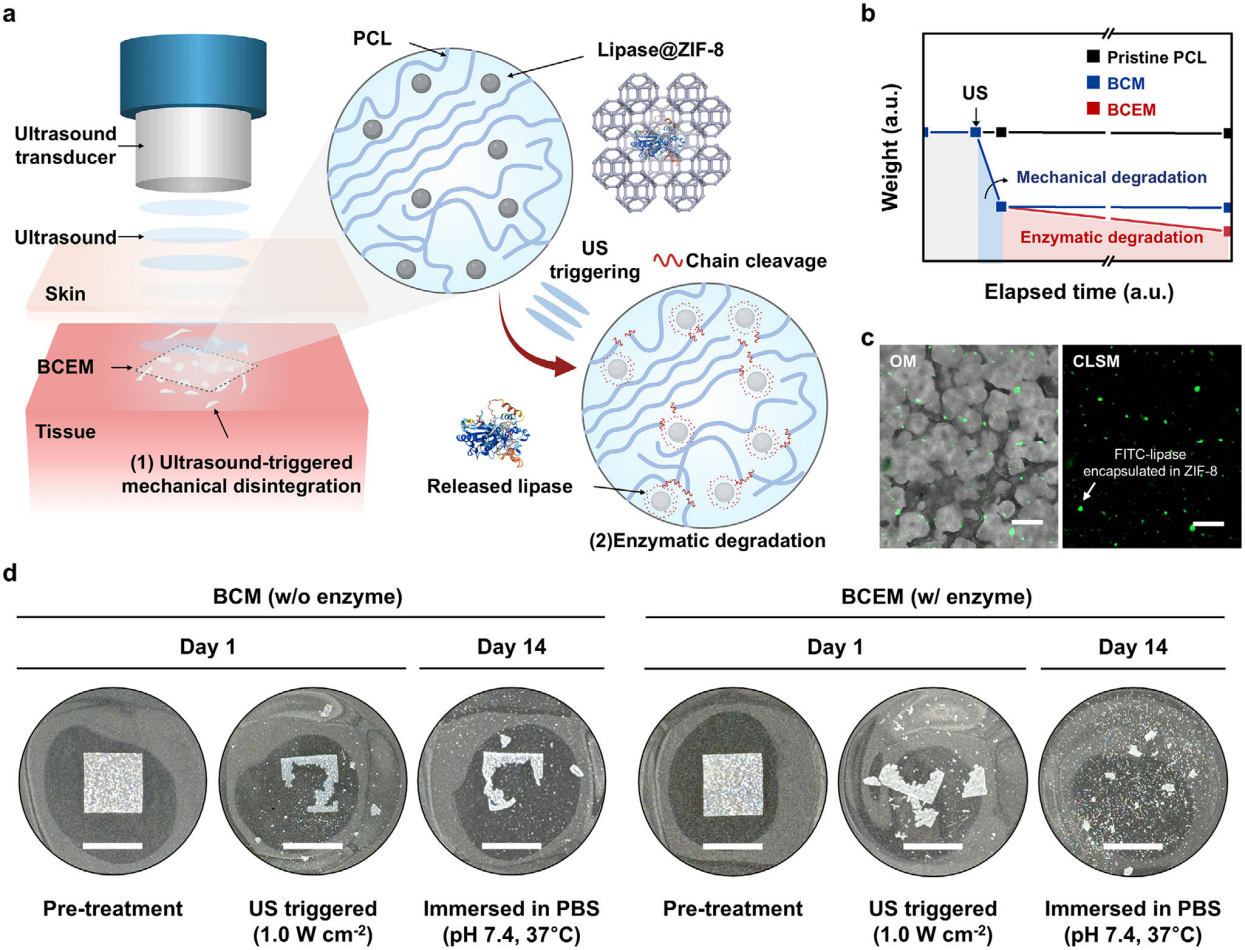
Overall concept of the SEA strategy and in vitro degradation results. (a) Schematic illustration of the cascadic degradation process. Created in BioRender. Kim, J. (2026) https://BioRender.com/63e0kcu. Lipase structure image reproduced/adapted from AlphaFold DB entry AF‐P26504‐F1 under CC BY 4.0. (b) Comparison of degradation rates among pristine bioresorbable materials, BCM, and BCEM. (c) Optical microscopy (OM) and confocal laser scanning microscopy (CLSM) images of BCEM composed of PCL and FITC‐lipase@ZIF‐8. (d) In vitro degradation profiles of BCM and BCEM. BCM exhibits ultrasound‐triggered mechanical degradation only (Day 1), with minimal subsequent particle size reduction (Day 14), whereas BCEM undergoes coupled ultrasound‐triggered mechanical and enzymatic degradation (Day 1), resulting in continuous fragment size reduction (Day 14).

However, certain MOFs can undergo structural collapse without pH variations when exposed to ions or molecules that competitively disrupt metal‐ligand coordination [26]. A representative example is ZIF‐8, which can be degraded in vivo under physiological conditions (pH 7) by phosphate ions, present in body fluid [27, 28]. Therefore, we designed BCEM consisting of PCL embedded with lipase@ZIF‐8, demonstrating that encapsulated lipase can be released from collapsed ZIF‐8 and subsequently catalyze the hydrolysis reaction, degrading the ester bonds of PCL. Figure 1a illustrates the demonstration of our SEA strategy degrading BCEM. Upon ultrasound triggering, PCL is mechanically disintegrated due to the acoustic impedance mismatch between PCL and lipase@ZIF‐8. Then, lipase is released from ZIF‐8 and initiates enzymatic degradation.

Figure 1b compares the degradation behavior of different systems. Pristine PCL remains intact under ultrasound without mechanical disintegration due to the absence of embedded lipase@ZIF‐8. Bioresorbable composite embedded with pristine ZIF‐8 (BCM) undergoes mechanical disintegration under ultrasound due to acoustic impedance mismatch, but the absence of lipase prevents further degradation of fragments. In contrast, BCEM exhibits cascadic mechanical to enzymatic degradation, resulting in the most accelerated degradation rate. Optical and confocal microscopy images of BCEM clearly show that lipase is localized within ZIF‐8 (Figure 1c). Lipases were labeled with fluorescein isothiocyanate (FITC) and encapsulated into ZIF‐8 (Figures S2–S4). These results confirm that ZIF‐8 functions as a nanocarrier, effectively isolating lipases from premature reactions with the surrounding PCL.

Figure 1d shows the in vitro degradation test results (phosphate‐buffered saline (PBS), pH 7.4, 37°C) (Figure S5). Without ultrasound excitation, pristine PCL, BCM, and BCEM exhibited only gradual and limited degradation over 14 days (Figure S6). Consistent with the concept in Figure 1b, BCEM degraded most efficiently. Pristine PCL remained intact under ultrasound (Figure S7). BCM was mechanically disintegrated by ultrasound, forming large fragments, but showed no further degradation due to the absence of an enzyme. In contrast, BCEM generated by ultrasound was further degraded into smaller particles through enzyme activity. As the amount of encapsulated and released enzyme is relatively low, the reaction may occur very slowly or appear almost negligible; however, by comparing the BCEM group with the pristine PCL and BCM groups, enzymatic activity was confirmed. To quantitatively support this observation, weight‐loss measurements were performed on the residual fragments (n = 3), which showed that BCEM exhibited approximately 15.0% greater mass loss than BCM after 2 weeks based on the mean values (Figure S8).

### 2.2 Mechanism and Demonstration of Lipase Release from ZIF‐8

**FIGURE 2:**
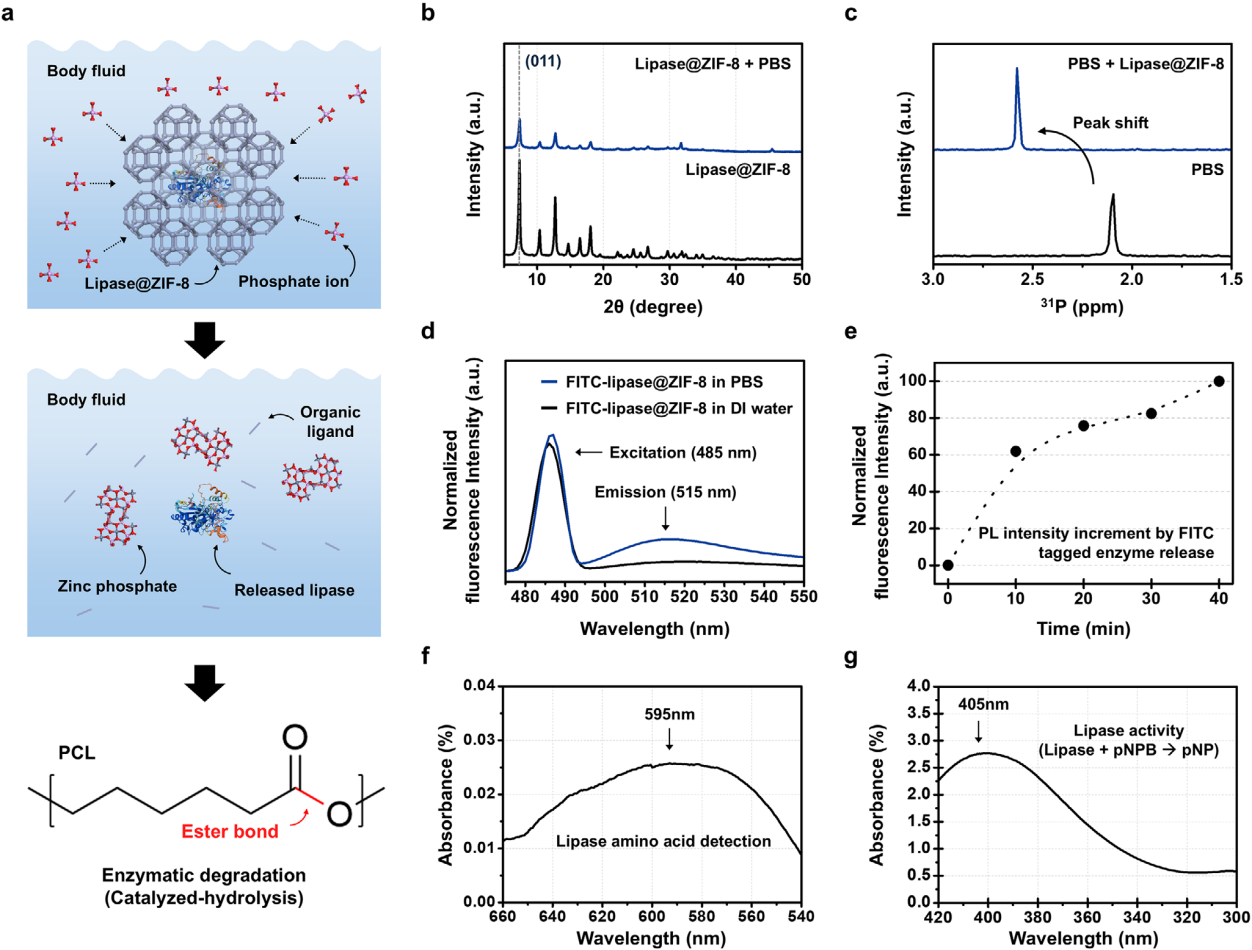
Mechanism and experimental proofs of lipase release from ZIF‐8. (a) Schematic illustration of the degradation and subsequent lipase release process of lipase@ZIF‐8. Lipase structure image reproduced/adapted from AlphaFold DB entry AF‐P26504‐F1 under CC BY 4.0. (b) XRD patterns displaying the gradual decrease in crystallinity of lipase@ZIF‐8 in PBS. (c) ^3^ ^1^P‐NMR spectra showing changes in the electronic environment of phosphorus atoms induced by lipase@ZIF‐8. (d) PL intensity comparison between FITC‐lipase@ZIF‐8 dispersed in DI water (without phosphate ions) and in PBS (with phosphate ions), tracking FITC‐lipase release. (e) Time‐dependent PL intensity of FITC‐lipase@ZIF‐8 immersed in PBS, monitoring the progressive release of FITC‐lipase. (f) UV–vis spectrum of the Bradford assay displaying a characteristic 595 nm absorption peak, confirming lipase detection. (g) UV–vis spectrum of the pNPB hydrolysis reaction displaying a distinct 405 nm absorption peak, evidencing the preserved catalytic activity of the released lipase.

Figure 2a depicts the enzyme release mechanism of ZIF‐8 in body fluids. When ZIF‐8 encounters phosphate ions (PO_4_^3–^), competitive binding occurs between zinc ions (Zn^2+^) and phosphate ions, forming zinc phosphate [Zn_3_(PO_4_)_2_] [27]. We hypothesized that enzymes can be released and demonstrated experimentally. X‐ray diffraction (XRD) measurement (Figure 2b) and phosphorus‐31 nuclear magnetic resonance (^31^P‐NMR) spectroscopy (Figure 2c) analyses demonstrate that the lipase@ZIF‐8 was degraded under the in vitro circumstances (pH 7.4 PBS, 37°C). After 40 min immersed in PBS, XRD showed decreased intensity of the (011) plane peak, indicating framework destruction. In parallel, ^31^P‐NMR revealed a peak downfield shift (0.48 ppm) caused by changes in the electronic environment of phosphorus atoms, consistent with the formation of zinc phosphate [27]. Next, lipase release was confirmed by photoluminescence (PL). First, FITC‐lipase@ZIF‐8 was immersed in deionized (DI) water (without phosphate ions) and PBS, respectively. The PL intensity at ∼515 nm was measured after 40 min, and normalized data showed that PL intensity increased significantly in PBS, while little change was observed in DI water (Figure 2d). This result proves the effect of phosphate ions on the structural collapse of lipase@ZIF‐8 and subsequent enzyme release. Second, the PL intensity of the PBS solution was tracked over time, and a gradual increase was found, indicating the release of enzymes (Figure 2e and Figure S9).

To further confirm the presence and activity of the released enzymes, Bradford assay and p‐nitrophenyl butyrate (pNPB) hydrolysis tests were conducted, respectively. Lipase@ZIF‐8 was immersed in PBS (pH 7.4, 37°C) for 40 min, and the supernatant was collected for analysis. First, the Bradford assay was performed. Upon reacting the supernatant with Coomassie Brilliant Blue G‐250 dye, a characteristic absorption peak at 595 nm appeared in the ultraviolet–visible (UV–vis) spectrum. This peak arises from the binding interaction between amino acid residues of the enzyme and the G‐250 dye, confirming the presence of the released lipase (Figure 2f and Figure S10). Next, the pNPB hydrolysis assay was carried out. When the collected supernatant was mixed with pNPB, the solution color changed from transparent to yellow, showing a distinct absorption peak at 405 nm, corresponding to p‐nitrophenol (pNP), the byproduct of enzymatic hydrolysis of pNPB (Figure 2g and Figure S11). Because the degradation mechanism of pNPB is hydrolysis, identical to that of PCL, these results verify the enzymatic activity of the released enzyme.

### 2.3 Mechanism of Ultrasound‐Induced Mechanical Disintegration in BCEM

**FIGURE 3:**
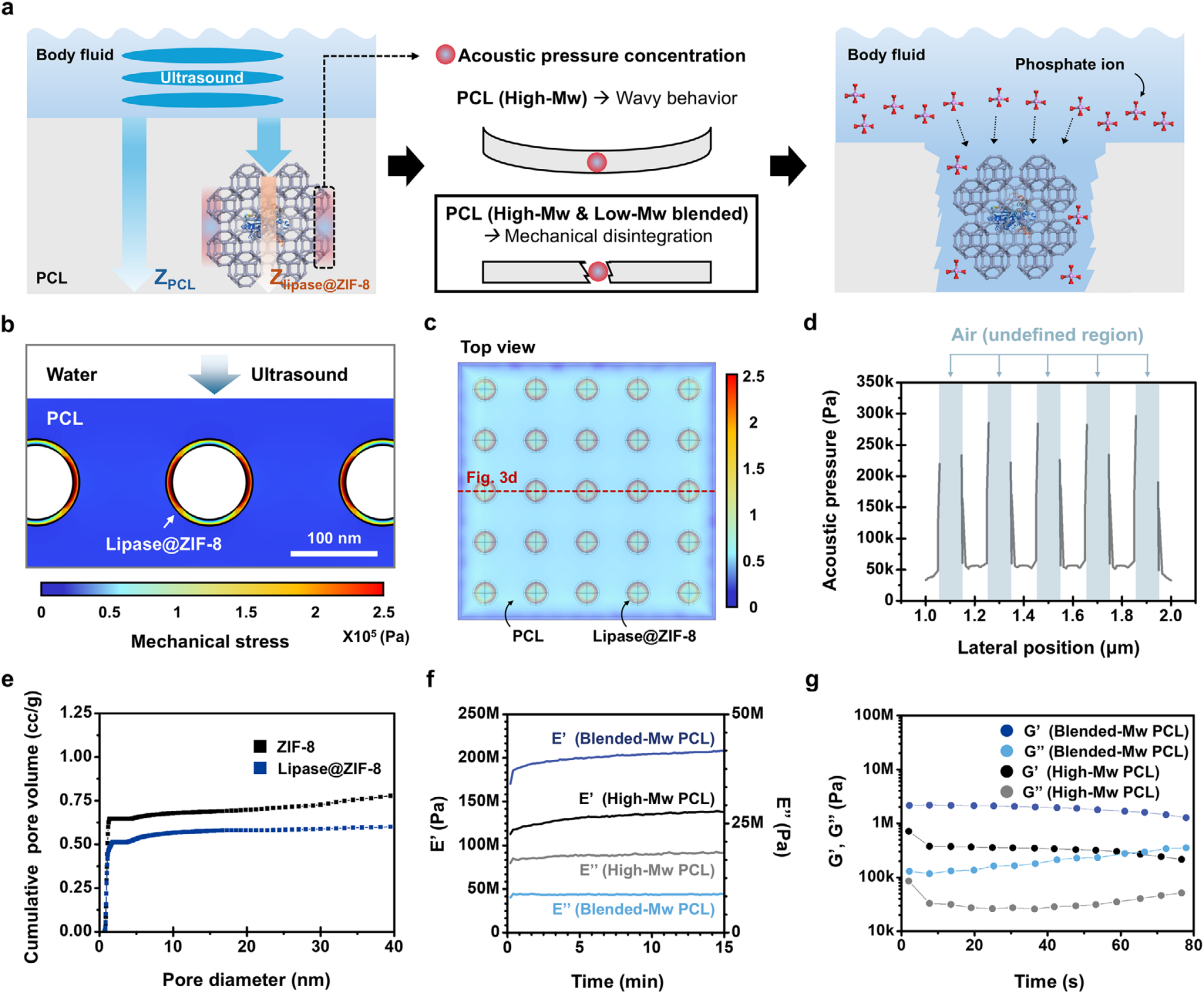
Mechanism of ultrasound‐induced disintegration of BCEM. (a) Schematic illustration of the overall degradation process, including the modulation of the molecular weight of PCL leading to mechanical disintegration. Lipase structure image reproduced/adapted from AlphaFold DB entry AF‐P26504‐F1 under CC BY 4.0. (b, c) COMSOL simulations showing the concentration of acoustic pressure at the interface between the PCL and the embedded lipase@ZIF‐8. (d) Quantitative representation of the acoustic pressure distribution corresponding to (c). (e) BET analysis presenting the cumulative pore volumes of ZIF‐8 and lipase@ZIF‐8, reflecting the internal air fraction. (f) DMA time‐sweep results showing the storage and loss moduli of the high‐ and blended‐Mw PCL. (g) Initial linear region of the rheometer time‐sweep results, displaying the intrinsic shear storage and loss moduli of the high‐ and blended‐Mw PCL.

Figure 3a illustrates the mechanism of mechanical disintegration of BCEM under ultrasound. Upon ultrasound triggering, the acoustic impedance mismatch between the PCL and lipase@ZIF‐8 leads to acoustic pressure concentration at the interface. However, high molecular weight (Mw) PCL polymers tend to dissipate acoustic energy and exhibit wavy behavior owing to their ductile property from chain entanglement. Therefore, to facilitate mechanical disintegration at the acoustic pressure concentration point, we blended high‐ with low‐Mw PCL, reducing chain entanglement compared to high‐Mw PCL alone. It has been reported that nearly unentangled networks show little fracture toughness, owing to the lack of stress dissipation pathways [29]. We optimized the blending ratio of high‐ and low‐Mw PCL to make BCEM to be mechanically disintegrated under the intended ultrasound triggering condition (1.0 W cm^−2^, 15 min) (Figure S12).

Figure 3b,c shows COMSOL simulation results, demonstrating acoustic pressure concentration at the BCEM interface under ultrasound by acoustic impedance mismatch between PCL and lipase@ZIF‐8 (Table S1). The acoustic impedances of PCL and lipase@ZIF‐8 were calculated to be 3.99 × 10^5^ and 2.31 × 10^6^ Rayl, respectively, where lipase@ZIF‐8 exhibits an acoustic impedance approximately 5.8 times higher than that of PCL. Quantified data from Figure 3c are summarized in Figure 3d. The solid mechanics module in COMSOL was applied, with the air inside the lipase@ZIF‐8 treated as an undefined region. The pressure sharply increases within the interface of the BCEM matrix and lipase@ZIF‐8, reaching 300–350 kPa, indicating strong acoustic impedance concentration. BCM and BCEM maintained comparable fracture forces to pristine PCL, indicating that MOF encapsulation did not significantly weaken the bulk mechanical integrity of the composites (Figure S13). The presence of air‐filled pores and assumption as undefined regions within lipase@ZIF‐8 was supported by BET analysis, as shown in Figure 3e. The cumulative pore volume distribution analyzed by the DFT method shows that pristine ZIF‐8 exhibits a higher pore volume (∼0.7–0.8 cc g^−1^), while lipase@ZIF‐8 displays a reduced value (∼0.5–0.6 cc g^−1^) due to partial filling of pores by encapsulated lipases. Nevertheless, both samples retain substantial internal void space, supporting the conclusion that the interior can reasonably be considered as air‐filled.

The storage modulus (E′) and loss modulus (E″) of PCL blends were evaluated by dynamic mechanical analysis (DMA) (Figure 3f). The E″/E′ ratio shows a damping property, which is the mechanical energy dissipating capability by converting to heat [30]. The higher E″/E′ ratio was shown in high‐Mw PCL, indicating relative viscous behavior and high damping capacity, while blended PCL showed a lower E″/E′ ratio, indicating relative elastic behavior and low damping capacity. Consistent trends were confirmed by rheometer‐based measurements of shear storage modulus (G′) and loss modulus (G″) (Figure 3g). The DMA and rheometer results clearly demonstrate the reduced damping properties of blended PCL arising from decreased chain entanglement. Consequently, by utilizing blended‐Mw PCL, the limited ultrasound energy dissipation occurred under acoustic pressure concentration, which facilitated mechanical disintegration.

### 2.4 In Vivo Demonstration of BCEM Degradation and Biocompatibility Evaluation

**FIGURE 4:**
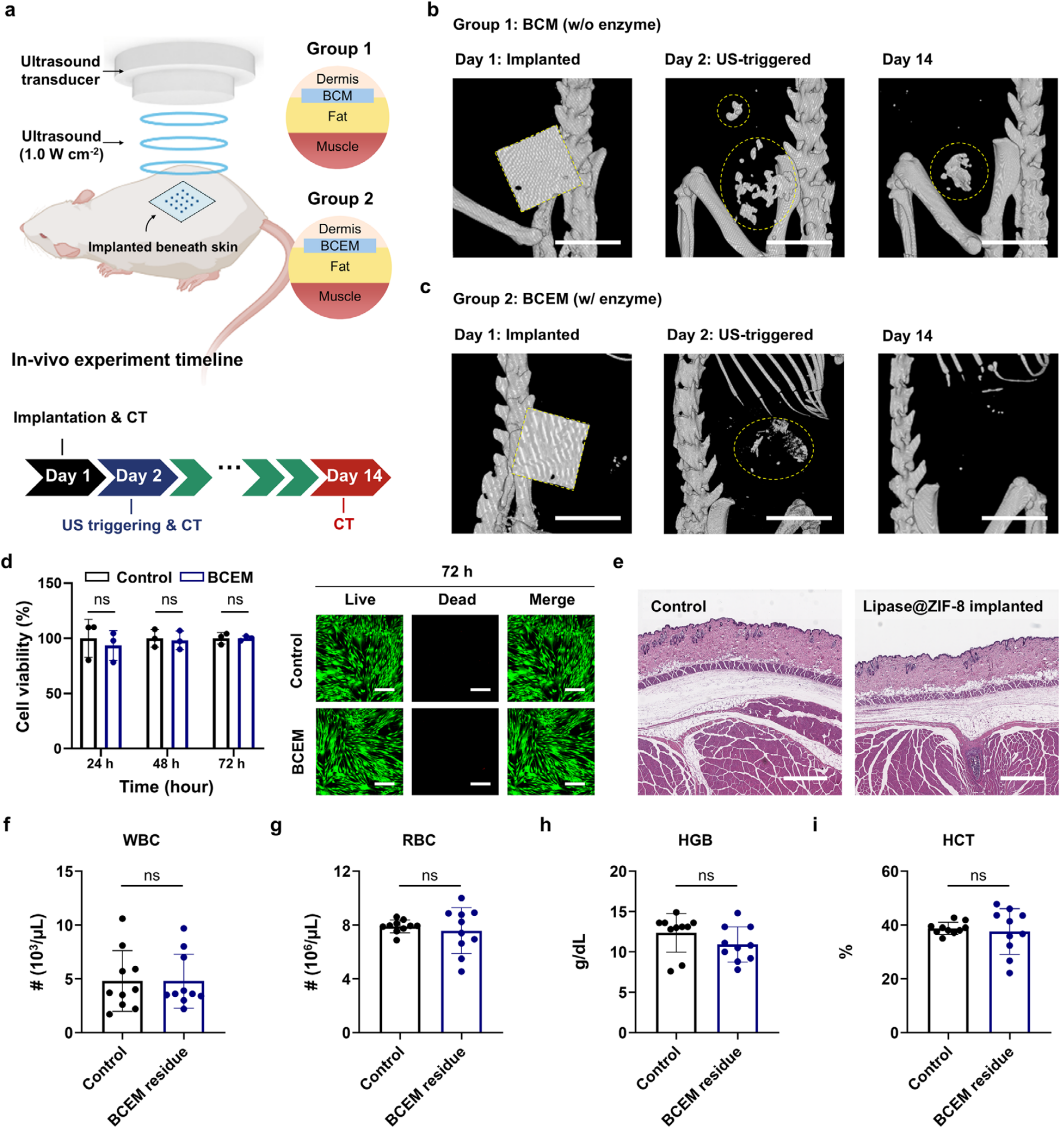
In vivo degradation results and biocompatibility assessments. (a) Schematic illustration of the in vivo experimental setup and the timeline of CT imaging. Created in BioRender. Kim, J. (2026) https://BioRender.com/k4b2lld. (b, c) Micro‐CT images of the BCM (b) and BCEM (c) groups, respectively. BCM exhibits ultrasound‐triggered mechanical fragmentation (Day 2), after which the fragmented residues remain over time (Day 14), whereas BCEM undergoes coupled mechanical disintegration and subsequent enzymatic degradation (Day 2), leading to a gradual reduction of residual fragments to a barely detectable level in vivo (Day 14). (d) Cell viability (n=3 per group) and live/dead assay results of the control group and BCEM group over 72 h. Data are presented as mean ± SD. (e) H&E staining images of the control group and lipase@ZIF‐8 group. (f–i) CBC results (n=10 per group) of BCEM residues, including WBC (f), RBC (g), HGB (h), and HCT (i) parameters. Data are presented as mean ± SD. *P*‐values in (d) and (f–i) are evaluated through a two‐sided *t*‐test; ns = non‐significant.

Figure 4a presents the schematic illustration of the in vivo degradation setup and computed tomography (CT) imaging timeline. Gold (Au) deposited (100 nm) BCEM films were implanted subcutaneously in three mice for micro‐CT imaging to track in vivo degradation (Figure S14). CT imaging was performed at three time points: BCEM implantation, ultrasound triggering, and 14 days after ultrasound triggering. The same procedure was carried out for the BCM group as a control. CT imaging revealed distinct differences between the two groups (Figure 4b,c). Both BCM and BCEM groups underwent mechanical disintegration under ultrasound (1.0 W cm^−^^2^, 15 min). However, after 14 days, fragments of the BCM group remained detectable by CT, whereas almost no particles were visible in the BCEM group. As observed in the in vitro experiments, fragments of BCEM were expected to remain; however, enzymatic degradation reduced the size of BCEM fragments, such that the detectable intensity of the deposited Au within each CT pixel (2 µm) decreased to a level close to the background signal. As the signal is low, resolution problems arise that residual particles may appear absent. Comparing the CT images between the BCM and BCEM groups, the enzymatic degradation was confirmed.

Biocompatibility of BCEM and its components was further validated through in vitro and in vivo assays. Figure 4d shows the cell viability results, where both control and BCEM groups maintained above 90% viability at 24, 48, and 72 h, confirming the absence of cytotoxicity (Figure S15). Representative live/dead fluorescence images after 72 h revealed predominantly live (green) cells and negligible dead (red) cells, verifying that BCEM does not impair cell compatibility. BCEM residues degraded by sonoenzymatic activation also showed no noticeable cytotoxicity, further indicating their biocompatibility (Figure S16). As PCL has been well characterized to undergo resorption via the citric acid cycle over time [31], histological analysis (H&E staining) was performed for lipase@ZIF‐8. Compared to controls, tissues collected after 14 days from the lipase@ZIF‐8 implant group exhibited normal morphology without signs of inflammation. Our results correspond to previous studies that organic ligands have previously been reported to be excreted from the body within 24 h [32], and zinc phosphate is a well‐known FDA‐approved material that gradually dissolves in vivo. These confirm that lipase@ZIF‐8 does not elicit significant adverse tissue reactions, demonstrating their biocompatibility (Figure S17).

To confirm the biosafety of total residues generated from BCEM generated by the SEA strategy, a complete blood count (CBC) was conducted. Compared with the control group (n = 10), the BCEM residue group (n = 10) exhibited nearly identical blood composition profiles after 14 days (Figure S18). White blood cell (WBC), a key marker of inflammation and immune activation, remained unchanged, indicating that generated BCEM residues did not trigger significant systemic inflammatory responses (Figure 4f). Similarly, red blood cell (RBC) (Figure 4g), hemoglobin (HGB) (Figure 4h), and hematocrit (HCT) (Figure 4i) values, which collectively reflect oxygen transport capacity and overall hematological balance, were also consistent between groups, confirming that BCEM residues did not impair hematological function.

### 2.5 Proof‐of‐Concept Demonstration with UB‐TENG

**FIGURE 5:**
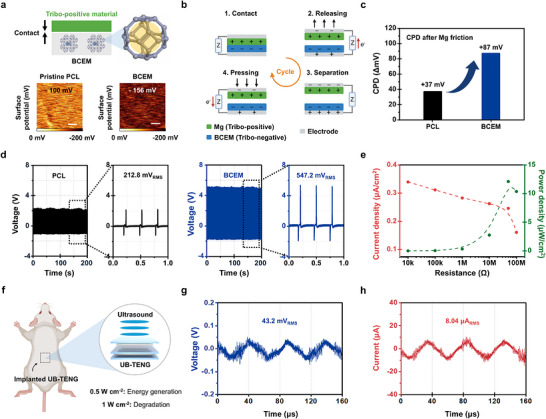
Triboelectric property of BCEM and application to UB‐TENG. (a) Schematic illustration and KPFM results showing the enhanced electronegativity of lipase@ZIF‐8 attributed to its internal surface area. Lipase structure image reproduced/adapted from AlphaFold DB entry AF‐P26504‐F1 under CC BY 4.0. (b) CPD comparison between pristine PCL and BCEM after friction with Mg. Created in BioRender. Kim, J. (2026) https://BioRender.com/8myt73q. (c) Schematic illustration of the energy generation mechanism of the TENG. (d) Pushing test (1 kgf, 10 min) showing the electrical outputs of pristine PCL and BCEM upon contact with Mg. The samples were fabricated with dimensions of 2 cm × 2 cm. (e) Power density measurements of BCEM under different applied loads. (f) Schematic illustration of the UB‐TENG structure and its implantation in a mouse. Created in BioRender. Kim, J. (2026) https://BioRender.com/zop9532. (g, h) In vivo voltage (g) and current (h) outputs generated under ultrasound application (0.5 W cm^−^^2^).

To demonstrate that our BCEM can function effectively throughout the therapeutic period and undergo degradation at the desired time, it was applied to an ultrasound‐driven triboelectric nanogenerator (TENG). Leveraging the deep tissue penetration capability of ultrasound, ultrasound‐powered TENGs have recently been actively explored as bioelectronic medicines, offering an alternative to conventional pharmacological approaches [33, 34]. First, the triboelectric properties of BCEM were investigated. (Figure 5a). When BCEM contacts a highly triboelectric positive material such as Mg, the BCEM exhibits triboelectric negative behavior, which was further reinforced by the embedded ZIF‐8 through the charge trapping effect due to the large internal area [35]. There are also reports that the enhanced output is attributed to increased surface roughness induced by ZIF‐8 embedding, which effectively enlarges the contact area between the triboelectric layers [36]. To analyze triboelectric properties, surface potential was measured using Kelvin probe force microscopy (KPFM), which provides direct evidence of triboelectric performance [37]. Results showed that pristine PCL had a potential of −100 mV, whereas BCEM exhibited −156 mV, confirming its stronger triboelectric negative property. Moreover, the contact potential difference (CPD) after friction with Mg increased to +87 mV for BCEM compared with +37 mV for pristine PCL, indicating greater electron acquisition capability (Figure 5c and Figure S19).

Figure 5b illustrates the working mechanism of the TENG device, where BCEM serves as the triboelectric negative material and Mg as the triboelectric positive material. Contact electrification at their interface induces electrostatic charges, driving current flow through the external circuit [38]. During the contact stage, the tribo‐positive Mg layer and the tribo‐negative BCEM layer come into contact, generating opposite charges on their surfaces. Upon release and subsequent separation, the induced potential difference drives electron flow through the external circuit. When pressed again, the two layers re‐establish contact, reversing the electron flow and completing the cycle. Through repeated contact‐separation cycles, continuous alternating current is generated, with the enhanced output attributed to embedded lipase@ZIF‐8. A pushing test (1 kgf, saturation time 10 min) demonstrated that BCEM generated significantly higher voltage output (547.2 mV_RMS_) compared with pristine PCL (212.8 mV_RMS_) (Figure 5d and Figure S20). Under the same conditions, current density and power density were also measured as a function of external load resistance, revealing a maximum power density of ∼12–13 µW cm^−^^2^ at 50 MΩ (Figure 5e).

Finally, an ultrasound‐driven, BCEM‐based TENG (UB‐TENG) was fabricated by adopting a double‐electrode‐mode device structure [39]. BCEM was utilized as both the encapsulation and triboelectric negative layers, with Mg serving as the triboelectric positive layer and electrode (Figure S21). In vitro electrical measurements confirmed that the UB‐TENG generated an electrical output of 227.4 mV_pp_ and 52.78 mV_RMS_ upon ultrasound application (20 kHz, 0.5 W cm^−^^2^). After the ultrasound‐triggering event (1.0 W cm^−^^2^ for 15 min), the triboelectric output was no longer observed, indicating that device operation ceased as a result of the cascaded degradation process (Figure S22). Figure 5f shows the schematic illustration of the in vivo experiment setup, where the UB‐TENG generated a triboelectric voltage of 122 mV_pp_ and 43.2 mV_RMS_ (Figure 5g), while a current of 22.75 µA_pp_ and 8.04 µA_RMS_ (Figure 5h), under ultrasound operation at 0.5 W cm^−^^2^. Upon increasing ultrasound intensity to 1.0 W cm^−^^2^, the UB‐TENG ceased energy generation and underwent the cascaded degradation process, confirming its on‐demand degradability.

## 3 Conclusions

In summary, we establish a SEA materials strategy for on‐demand, non‐invasive elimination of bioresorbable systems. By integrating ultrasound‐triggered mechanical disintegration with enzyme‐catalyzed hydrolysis, SEA enables cascading degradation that bridges macroscopic fragmentation and molecular‐level decomposition. In vitro and in vivo studies demonstrate rapid and controllable degradation of BCEM under medically relevant ultrasound (1.0 W cm^−^^2^, 15 min), while comprehensive biocompatibility assessments confirm the biosafety of the degradation products. As a proof‐of‐concept, a UB‐TENG exhibits stable output under low‐intensity ultrasound, highlighting the programmable nature of this platform. Overall, this work addresses persistent challenges of incomplete and unpredictable degradation in bioresorbable materials and establishes a broadly applicable materials platform for clinically deployable transient biomedical devices.

## 4 Experimental Section/Methods

### 4.1 Synthesis of Lipase@ZIF‐8

Lipase@ZIF‐8 synthesis was performed by a co‐synthesis method [24, 25]. Briefly, separate solutions of zinc acetate dihydrate (0.293 g in 1 mL DI water) (Sigma Aldrich, Mw 219.51), 2‐methylimidazole (1.09 g in 4 mL DI water) (Sigma Aldrich, Mw 82.1), and lipase (5 mg in 1 mL DI water) (Sigma Aldrich, Amano lipase from Pseudomonas fluorescens) were prepared and mixed for 10 min at room temperature (RT, 300 rpm). The resulting white precipitate was centrifuged (10,000 rpm, 10 min), washed several times with DI water and ethanol each, then freeze‐dried. Pristine ZIF‐8 was synthesized by just excluding the lipase solution. Apparent encapsulation efficiency (EE) of lipase was calculated by the Bradford assay, comparing the supernatant after lipase@ZIF‐8 synthesis and the initial lipase solution (Table S2). For FITC‐labeled lipase encapsulation, lipase solution (3 mg in 1 mL carbonate buffer, pH 9.5) was mixed with FITC solution (8 mg dissolved in DMSO). The mixture was shaken in the dark at 300 rpm for 3 h at ambient temperature. Unbounded FITC was removed by dialysis, and the FITC‐labeled lipase was then encapsulated in ZIF‐8, following the same procedure as above.

### 4.2 BCEM Fabrication

PCL solutions were prepared by dissolving PCL granules in chloroform (0.1 g mL^−^^1^) (Samchun Chemical, 99.5% chloroform). Two types of PCL with different Mw (Mn 10,000 and Mn 80,000; Sigma–Aldrich) were mixed at an 8:2 ratio (Mn 10,000: Mn 80,000) and dissolved in chloroform. Freeze‐dried lipase@ZIF‐8 powders were finely ground, added to the PCL solution (0.04 g mL^−1^), and co‐stirred. Uniform‐thickness films were subsequently fabricated using a bar coater (DAO Technology, DAO‐CO02‐VH).

### 4.3 In Vitro Weight‐Loss Measurement

For quantitative weight‐loss measurements, six independent samples were prepared for each of the BCM and BCEM groups (2 cm × 2 cm × 40 µm). Three samples were weighed before and after ultrasound treatment to quantify the immediate weight loss induced by ultrasound triggering. The remaining three samples were weighed before ultrasound treatment and again after ultrasound treatment, followed by 2 weeks of incubation in PBS to quantify the cumulative weight loss. For ultrasound triggering, samples were placed in glass dishes containing 20 mL of PBS (Samchun Chemical, pH 7.4). Ultrasound was applied at an intensity of 1.0 W cm^−^^2^ for 15 min, with the ultrasound probe positioned less than 5 mm from the film surface. The residues were collected by vacuum filtration using a membrane filter with a 5 µm pore size and dried in an oven at 40°C before weighing. The weight loss was calculated as a percentage relative to the initial dry weight of each sample, and the data are presented as mean ± SD.

### 4.4 XRD and ^31^P‐NMR

Lipase@ZIF‐8 powders were immersed in PBS (Samchun Chemical, pH 7.4) (1 mg mL^−^^1^, 37°C, 40 min) and centrifuged (10,000 rpm, 10 min). The retrieved lipase@ZIF‐8 powders were analyzed by XRD (Rigaku, Ultima IV), and identical amounts of pristine and retrieved lipase@ZIF‐8 powders were compared. In addition, ^3^ ^1^P‐NMR (400 MHz, D_2_O) was conducted by an NMR spectrometer (Bruker, Avance III HD 400) using pristine PBS and PBS incubated with lipase@ZIF‐8. A 5 mL supernatant of each solution was collected and analyzed by NMR spectroscopy.

### 4.5 PL Measurement

FITC‐lipase@ZIF‐8 powders were immersed in DI water or PBS (1 mg mL^−^^1^, 37°C, 40 min) and kept in the dark to prevent photobleaching. Supernatants were collected into a four‐window cuvette and analyzed by a steady‐state spectrofluorometer (Edinburgh Instruments, FS5). In addition, for the PBS group, supernatants were collected at 10, 20, 30, and 40 min to monitor changes in PL intensity over time.

### 4.6 UV–Vis

Lipase@ZIF‐8 powders were immersed in PBS (1 mg mL^−1^, 37°C, 40 min). Pristine PBS and lipase@ZIF‐8 immersed PBS were analyzed using the Bradford assay. 2 mL of supernatant was collected from each solution, mixed with 2 mL of G250 reagent, and incubated for 5 min before transferring to a two‐window cuvette for absorbance measurement with a UV–vis spectrophotometer (Jasco, V‐650), checking at 595 nm. Furthermore, 4 mL of supernatant was collected from each solution and mixed with 40 µL p‐nitrophenol (pNPB) solution to check the activity of released lipase. The mixture was incubated at 37°C for 5 min, and the reaction was then analyzed by UV–vis spectroscopy at 405 nm.

### 4.7 DMA, Rheometer Measurement

PCL films with dimensions of 1 cm × 3 cm × 40 µm were prepared from both high‐ and blended‐ Mw PCL for DMA analysis (Hitachi, DMA7100) (1 Hz, RT, time‐sweep mode, 15 min). For rheometer analysis (TA instruments, HR‐30) (1 Hz, time‐sweep mode), PCL samples with a diameter of 3 cm with a thickness of 1 mm were also prepared from both high‐ and blended‐Mw PCL. In DMA, thinner films are required, as excessively thick specimens can reduce measurement accuracy, whereas overly thin films may produce weak signals and high noise. By contrast, rheometer analysis requires relatively thicker samples to ensure stable plate contact and uniform transmission of shear stress.

### 4.8 In Vivo Experiment

All studies on mice were approved by the Institutional Animal Care and Use Committee (IACUC) of Yonsei University (IACUC‐202509‐2126‐01). 6‐week‐old mice were anesthetized with 2–2.5% isoflurane via inhalation to induce general anesthesia. BCM and BCEM films were coated with Au 100 nm via an e‐beam evaporator (JVAC, J‐VACE‐7000) for CT‐imaging and inserted under the dermis of mice. The BCM group consists of three mice: an implantation‐only group (n = 1), an implantation & ultrasound‐triggered group (n = 1), and a 14‐day prolonged group following implantation and ultrasound triggering (n = 1). In the same way, the BCEM group also consists of three mice. Ultrasound was applied using a commercially available ultrasound transducer and generator (Mirae Ultrasonic, Mirae MV100) at an intensity of 1.0 W cm^−2^ for 5 min, 3 times repeating. Mice were sacrificed with CO_2_ gas, and CT images were acquired using micro‐CT (Bruker, Skyscan 1276). The images were post‐processed with CTAn and CTVox software for 3D visualization.

### 4.9 MTT Assay

For the cytotoxicity assay, cells were seeded in 96‐well plates at 1 × 10^4^ cells/well and incubated for 24, 48, and 72 h. Each well was treated with MTT solution (0.5 mg mL^−1^ in PBS) and left for 2 h at 37°C. The resulting formazan was dissolved in DMSO, and optical density was measured at 570 nm using a microplate reader. Data are presented as mean ± SD.

### 4.10 H&E Staining

2 mg of lipase@ZIF‐8 contents corresponding to ∼5 mg of BCEM film (1 cm × 1 cm × 40 µm) was implanted under the dermis of the experimental group mouse (n = 1), and tissue samples were collected after 2 weeks from the control group (n = 1) and experimental group. Tissue samples were fixed in 4% formalin, embedded in paraffin, and sectioned at 5 µm thickness. Sections were deparaffinized, rehydrated, and stained with hematoxylin for 5 min, followed by eosin for 1 min. After dehydration and mounting, stained tissues were examined under a light microscope.

### 4.11 CBC Test

Ultrasound‐triggered, mechanically disintegrated BCEM film residues (1 cm × 1 cm × 40 µm) were implanted under the dermis of the experimental group (n = 10), and blood samples were collected after 2 weeks from the control group (n = 10) and the experimental group (n = 10). Blood was collected in anticoagulant‐coated tubes and analyzed using a hematology analyzer (NIHON KOHDEN, MEK‐6550). Data are presented as mean ± SD.

### 4.12 Electrical Output Measurement

Voltage output was measured using an oscilloscope (Tektronix, DPO3052) equipped with a 40 MΩ probe (Tektronix, P5100A), while current output was recorded with a low‐noise current amplifier (FEMTO, DLPCA‐200) connected to the oscilloscope. A pushing tester (1 kgf, saturation time of 10 min) was employed to evaluate the enhancement of the triboelectric output of PCL by the incorporation of lipase@ZIF‐8, using the fabricated measurement samples. Current density and power density were further measured under the same conditions with different load resistances connected between the measurement sample and the current amplifier. In vitro UB‐TENG measurements were conducted by placing the UB‐TENG device in a glass dish containing 20 mL of PBS (pH 7.4). The ultrasound probe was positioned less than 5 mm from the device surface. For in vivo measurements, the UB‐TENG device was implanted subcutaneously under the dorsal skin of mice, and ultrasound gel was applied to ensure acoustic coupling during ultrasound application.

### 4.13 UB‐TENG Fabrication

The UB‐TENG (active area: 1 cm × 1 cm) was fabricated through side hot‐pressing and consisted of a BCEM layer (substrate), an Mg/BCEM layer (electrode/tribo‐negative layer), and an Mg/BCEM layer (tribo‐positive/encapsulation layer). Mg/BCEM layer was prepared by depositing Mg (100 nm) onto the BCEM by e‐beam evaporation (JVAC, J‐VACE‐7000).

### 4.14 Statistical Analysis

Statistical analyses were performed for the in vitro weight‐loss measurements (Figures S6 and S8), cell viability tests (Figure 4d and Figure S16), and complete blood count analyses (Figure 4f–i and Figure S18). For data pre‐processing, the in vitro degradation data were normalized to the initial weight of each sample, which was set as 100%. Cell viability data were normalized to the mean value of the control group at each corresponding time point, which was set as 100%. Complete blood count data were analyzed using the raw measured values without normalization. No outliers were excluded from the statistical analyses. All data are presented as mean ± SD, and the sample size (n) for each experiment is indicated in the corresponding figure captions. Statistical significance was assessed using a two‐sided *t*‐test with GraphPad Prism 8; ns, not significant; ^*^ *p* < 0.05.

## Conflicts of Interest

D.‐M.L., B.K., and S.‐W.K. are inventors on a Korean patent (KR 10‐2348997) and on a U.S. patent (US 11878190 B2), related to on‐demand bioresorbable triboelectric generators based on nanocomposites incorporating nanocarriers used in this work. The remaining authors declare no competing interests.

## Supporting information




**Supporting File**: adma73921‐sup‐0001‐SuppMat.docx.

## Data Availability

The data that support the findings of this study are available from the corresponding author upon reasonable request.
