# Supplementary material for: Sonoenzymatically Triggered Cascading Degradation of Bioresorbable Materials for On‐Demand Transient Triboelectric Implants
Source: Adv Mater. 2026 Jul 8;38(45):e73921. doi: 10.1002/adma.73921 (PMC13471871; doi:10.1002/adma.73921)
Supplement: Supplementary file 1 [file ADMA-38-e73921-s001.docx]

Supporting Information

Sonoenzymatically Triggered Cascading Degradation of Bioresorbable Materials for On-Demand Transient Triboelectric Implants

*Jinsong Kim^†^, Dong-Min Lee^†^, Youngwook Chung, Byung-Joon Park, Hyeon Mo, Jong Won Seon, Han-Yup Yum, Bosung Kim, Byung-Ok Choi, and Sang-Woo Kim^*^*

TABLE OF CONTENTS

Figure S1 | Enzyme activity evaluation

Figure S2 | Synthesis process photographs of the lipase@ZIF-8

Figure S3 | Free-FITC removal process photographs

Figure S4 | BCEM fabrication process

Figure S5 | In vitro degradation experiment setup

Figure S6 | Weight-loss measurements of BCM and BCEM without ultrasound application

Figure S7 | Photographs of pristine PCL before and after ultrasound triggering

Figure S8 | Weight-loss measurements of BCM and BCEM after ultrasound triggering

Figure S9 | PL measurement to track FITC tagged lipase release overtime

Figure S10 | Sample images of Bradford protein assay

Figure S11 | Sample images of pNPB hydrolysis experiment

Figure S12 | Tensile test results for high-Mw PCL and blended-Mw PCL

Figure S13 | Tensile test results of PCL, BCM, and BCEM

Figure S14 | Photograph of in vivo degradation experiment setup

Figure S15 | Live/Dead staining images of BCEM throughout three days of culture

Figure S16 | MTT assay of BCEM residues

Figure S17 | Full images of H&E staining test

Figure S18 | Complete blood count (CBC) test results

Figure S19 | KPFM measurement

Figure S20 | Sample preparation method and images for pushing test

Figure S21 | Double electrode structure of UB-TENG

Figure S22 | In vitro electrical output measurement of UB-TENG

Table S1 | COMSOL simulation parameters and acoustic impedance calculation

Table S2 | Apparent encapsulation efficiency

**Figure S1 | Enzyme activity evaluation.** Weight loss of PCL films (Mn 80,000) under presence of enzyme to show lipase activity. PCL films (2 cm × 2 cm × 40 μm) were incubated in phosphate buffer saline (PBS pH 7.4) with or without enzyme (1 mg/5 mL). In the absence of enzyme, almost no weight loss of PCL was observed, whereas in the presence of enzyme, the PCL mass decreased drastically, with most of it degraded within 24 h.

**Figure S2 | Synthesis process photographs of the lipase@ZIF-8.** Three separate solutions, lipase in DI water, 2-methylimidazole in DI water, and zinc acetate dihydrate in DI water, were mixed together, resulting in the formation of a suspension within approximately 20 sec.

**Figure S3 | Free-FITC removal process photographs.** FITC-tagged lipase was purified by subjecting the solution to repeated dialysis steps, in order to remove non-tagged FITC.

**Figure S4 |** **BCEM fabrication process.** PCL was dissolved in chloroform and mixed with lipase@ZIF-8 to prepare a solution, which was then cast onto a glass substrate. The film thickness was controlled using a bar coater, followed by solvent evaporation over time. Created in BioRender. Kim, J. (2026) https://BioRender.com/2ohd1xo

**Figure S5 | In vitro degradation experiment setup.** The distance between ultrasound probe and film immersed in PBS was set as < 5 mm.

**Figure S6 | Weight-loss measurements of BCM and BCEM without ultrasound application.** Both BCM (n=3) and BCEM (n=3) showed an initial decrease in weight during the early stage of incubation, which is likely attributable to the dissolution of exposed MOF particles on the PCL surface in PBS (pH 7.4). After this initial weight loss, the remaining weight was largely maintained, indicating that the MOF particles embedded within the PCL matrix were stably encapsulated and did not induce further substantial mass loss. In the case of BCEM, a slightly greater weight loss was observed compared with BCM, presumably because partial dissolution of the MOF allowed the release of encapsulated lipase, which promoted additional degradation of the PCL matrix. However, the weight difference between the BCM and BCEM groups at day 14 was not statistically significant under non-triggered conditions (ns, *P* = 0.0834), indicating limited enzyme-assisted degradation in PBS without ultrasound triggering. In contrast, the weight-loss experiment conducted after ultrasound triggering (Figure S8) showed a statistically significant difference between the BCM and BCEM groups (**P* = 0.0107). This result supports the sonoenzymatic cascading degradation behavior of PCL, where ultrasound-induced matrix disintegration activates additional enzyme-assisted degradation. Data in Figure S6 and S8 are presented as mean ± SD. *P*-values are evaluated through two-sided *t*-test; ns = non significant; **P* < 0.05;

**Figure S7 | Photographs of pristine PCL before and after ultrasound triggering.** Ultrasound treated result of pristine PCL film (blended-Mw, 2 cm × 2 cm × 40 μm). remained intact.

**Figure S8 |** **Weight-loss measurements of BCM and BCEM after ultrasound triggering.** The degradation behaviors of BCM and BCEM were quantitatively compared by measuring the remaining weight immediately after ultrasound treatment (20 kHz, 1.0 W cm^-2^) and after 14 days of incubation at PBS buffer (pH 7.4, 37 °C). For each condition, independent samples were used, and the data are presented as mean ± SD (n = 3). Although BCM and BCEM showed comparable weight loss immediately after ultrasound-induced mechanical disintegration, BCEM exhibited a significantly reduced weight after 2 weeks compared to BCM, with an approximately 15.0% difference in the mean values (*P* = 0.0107). *P*-values are evaluated through two-sided *t*-test; ns = non significant; **P* < 0.05;

**Figure S9 |** **PL measurement to track FITC tagged lipase release overtime.** The maximum intensities over 515 nm from the intensity data at each time point were summarized in Figure 2e.

**Figure S10 | Sample images of Bradford protein assay.** (Left) Baseline: PBS + G250 dye (Right) Supernatant (PBS + lipase@ZIF-8) + G250 dye.

**Figure S11 | Sample images of pNPB hydrolysis experiment.** (Left) Baseline: PBS + pNPB (Right) Supernatant (PBS + lipase@ZIF-8) + pNPB.

**Figure S12 | Tensile test results for high-Mw PCL and blended-Mw PCL.** Fracture toughness and ductility were lowered in blended-Mw PCL. The formulation containing only low-Mw PCL (10:0) failed to form a stable film, demonstrating that high-Mw PCL was required to preserve film-forming ability and mechanical integrity before activation. Furthermore, under our intended ultrasound condition of 1.0 W cm⁻², the MOF composites of 6:4 and 7:3 blend matrix did not undergo noticeable mechanical disintegration, whereas the MOF composite of 8:2 blend matrix was mechanically disintegrated after 15 min of ultrasound irradiation.

**Figure S13 | Tensile test results of PCL, BCM, and BCEM.** Compared with bare PCL, both BCM and BCEM exhibited a slight increase in Young’s modulus after MOF embedding. This result suggests that the incorporated MOF particles contributed to the restricted mobility and elongation of PCL chains. In other words, the MOF particles may act as physical rigid domains that hinder chain extension during tensile deformation. Despite this reduced deformability, the maximum force value of BCM and BCEM was comparable to that of bare PCL, indicating that MOF encapsulation did not significantly compromise the overall mechanical strength of the composite films.

**Figure S14 | Photograph of in vivo degradation experiment setup.**

**Figure S15 | Live/Dead staining images of BCEM throughout three days of culture.** (Scale bar: 200 μm).

**Figure S16 | MTT assay of BCEM residues.** BCEM residues (n=3) have been produced by sonoenzymatic activation (1.0 W cm^-2^, 15 min). Data are presented as mean ± SD. *P*-values are evaluated through two-sided *t*-test; ns = non significant;

**Figure S17 | Full images of H&E staining test.** (Scale bar: 600 μm).

**Figure S18 | Complete blood count (CBC) test results.** CBC test was conducted 2 weeks after implantation of BCEM residues in mice (n=10). (a) Mean Corpuscular Volume (MCV). (b) Mean Corpuscular Hemoglobin (MCH). (c) Mean Corpuscular Hemoglobin Concentration (MCHC). (d) Platelet count (PLT). Data are presented as mean ± SD. *P*-values are evaluated through a two-sided t-test; ns = non significant.

**Figure S19 | KPFM measurement.** (a) Surface potential of PCL increased from -100 mV to -63 mV (CPD = +37 mV). (b) Surface potential of BCEM increased from -156 mV to -69 mV (CPD = +87 mV) after Mg friction. (c) Surface potential of BCM increased from -133 mV to -60 mV (CPD = +73 mV) after Mg Friction (Scale bar: 1 μm).

**Figure S20 | Sample preparation method and images for pushing test.** (Scale bar: 2 cm).

**Figure S21 | Double electrode structure of UB-TENG.**

**Figure S22 | In vitro electrical output measurement of UB-TENG.** (a) Under 0.5 W cm^-2^ ultrasound condition, UB-TENG generated stable output of 52.78 mV_RMS_, exhibiting a clear sinusoidal AC voltage waveform, which originated from contact–separation between the Mg layer (tribo-positive) and the BCEM layer (tribo-negative). (b) After the triggering event (1.0 W cm⁻², 15 min), the upper encapsulation layer composed of BCEM was mechanically disintegrated, resulting in the loss of the regular AC output.

|  | Density  (kg/m^3^) | Young’s modulus  (MPa) | Poisson’s ratio | Acoustic impedance  (Rayl) |
| --- | --- | --- | --- | --- |
| MOF | 950 | 5600 | 0.4 | 2.31×10^6^ |
| PCL | 1145 | 139 | 0.45 | 3.99×10^5^ |

**Table S1 | COMSOL simulation parameters and acoustic impedance calculation.** Reference values of density, Young’s modulus, and Poisson’s ratio are used in Figure 3b [1-4]. Measured storage modulus (E′) of 139 MPa for blended PCL was used as an approximation for Young’s modulus (E). Acoustic impedance, defined as the ratio of acoustic pressure to particle velocity in a medium, is a key parameter for characterizing the ultrasound transmission properties of materials [5]. Acoustic impedance can be estimated from the Young’s modulus and density of the medium, as follows:

$$Z= \rho\cdot v= \sqrt{E\cdot\rho}$$

where, Z represents the acoustic impedance (unit: kg m^−2^ s^−1^), v represents the speed of sound in a specific medium (unit: m s^−1^), ρ represents the density of the medium (unit: kg m^−3^), and E represents the Young’s modulus of the medium (unit: kg m^−1^ s^−2^). For PCL, the Young’s modulus and density are 139 MPa and 1,145 kg m^−3^, respectively, giving an acoustic impedance of 3.99×10^5^ Rayl. Similarly, for lipase@ZIF-8, the Young’s modulus and density values of pristine ZIF-8 were adopted as approximate parameters. The Young’s modulus and density are 5600 MPa and 950 kg m^−3^, respectively, giving an acoustic impedance of 2.31×10^6^ Rayl.

|  | Absorbance (A) |
| --- | --- |
| Supernatant + G250 | 0.090567 |
| Lipase solution + G250 | 0.477798 |

**Table S2 | Apparent encapsulation efficiency.** To estimate apparent encapsulation efficiency (EE), the absorbance(A) at 595 nm of the supernatant was measured using the Bradford assay and compared with that of the initial lipase solution.

Apparent EE (%) = (1 – A_Supernatant + G250_ / A_Initial lipase solution + G250_) × 100 = 81.04 %

**References**

1.	S. Eslava, L. Zhang, S. Esconjauregui, et al., “Metal-Organic Framework ZIF-8 Films As Low-κ Dielectrics in Microelectronics,” *Chemistry of Materials* 25(1), 27-33(2012).

<https://doi.org/10.1021/cm302610z>

2.	M. Erkartal and M. Durandurdu, “Pressure-induced amorphization, mechanical and electronic properties of zeolitic imidazolate framework (ZIF-8),” *Materials Chemistry and Physics* 240, 122222(2020).

<https://doi.org/10.1016/j.matchemphys.2019.122222>

3.	K.S. Tiaw, S.W. Goh, M. Hong, Z. Wang, B. Lan, and S.H. Teoh, “Laser surface modification of poly(ε-caprolactone) (PCL) membrane for tissue engineering applications,” *Biomaterials* 26(7)*,* 763-769(2005).

<https://doi.org/10.1016/j.biomaterials.2004.03.010>

4.	P. Zhang, Z. Gao, Q. Zhang, A. Khattab, and G. Li, “Fracture Behavior Characterization of Arcan Polycaprolactone Based Polymer Composites Prepared by Polymerization Induced Phases Separation,” *Polymer composites* 40(3), 1198-1208(2018).

<https://doi.org/10.1002/pc.24831>

5.	D.-M. Lee, M. Kang, I. Hyun, et al., “An on-demand bioresorbable neurostimulator,”

*Nature Communications* 14, 7315(2023).

<https://doi.org/10.1038/s41467-023-42791-5>
